# New Technique of Single-Point Scleral Fixation of the Smaller-Incision New-Generation Implantable Miniature Telescope with an 18-Month Follow-Up Period

**DOI:** 10.3390/life16020274

**Published:** 2026-02-05

**Authors:** Agnieszka Nowosielska, Grzegorz Rotuski

**Affiliations:** Warsaw Eye Hospital, 01-258 Warsaw, Poland

**Keywords:** age-related macular degeneration, geographic atrophy, implantable ophthalmic microtelescope, SING IMT, cataract surgery

## Abstract

Background: The implantable miniature telescope is used to provide functional vision for patients with advanced AMD. However, despite the considerable cost of the device, there are strict criteria to be met for this procedure, since the patients require challenging neuroadaptation afterward, which sometimes fails and leads to the necessity of device explantation. Visual outcomes also depend on the stability of the microtelescope; tilts cause unwanted optical aberrations and can lead to device luxation, with sight-threatening complications. Case report: This case presents a novel technique for fixing the ophthalmic telescope device SING-IMT™. A 76-year-old female with pre-operative visual acuity of 15 letters on the ETDRS scale underwent surgery on her left eye. The superior haptic was fixed at the 12 o’clock position with a Prolene 5-0 suture, achieving good postoperative stability. The implant was stable throughout the entire observation period. Conclusions: Implant stability is crucial for maximizing visual potential in patients with advanced AMD selected for the procedure, since visual acuity in the peripheral retina, where the perceived image eventually lands, is much lower than the macula. Therefore, there is a need to standardize surgical approaches and use objective follow-up measures to assess long-term patient satisfaction.

## 1. Introduction

Several surgical approaches have been used in order to improve visual acuity in patients with advanced age-related macular degeneration (AMD). Nonetheless, the pursuit of the optimal cure is still ongoing and significantly challenging. So far, trials to replace the damaged central retina with a bioelectrical implant or organoid structure have not yet brought functional central vision [1,2].

SING IMT is a small implantable telescope designed to improve visual acuity in patients with severely impaired visual acuity due to dry and wet AMD [3]. It is a second-generation Galilean telescope that provides a magnification of 2.7×, projecting the perceived image to the part of the retina surrounding the injured macula region. The main part of the device is a 3.6 mm optical component made of Quartz glass, enclosed in a silicone tube, for a total diameter of 10.8 mm, according to the manufacturer’s details. It is designed to be stabilized in the posterior capsule by three square silicone haptics and should be implanted after successful crystalline lens removal. The IMT telescope has a higher weight than a regular IOL—in typical air conditions, it is around 120 mg, but this value is halved when suspended in the aqueous conditions inside the eye. Implantation of this device requires a larger globe opening than is performed during regular small-incision cataract surgery.

Inclusion criteria for patients recommended by the producer are strict and include age over 55 years, stable visual acuity loss resulting from bilateral advanced AMD, a phakic target eye, and good peripheral vision in the fellow eye. On ophthalmic examination, choroidal neovascularization, intraocular or corneal surgery, corneal stromal or endothelial dystrophy, glaucoma, steroid high-responsiveness, and a refractive error of <−6.00 D or >+4.00 D need to be excluded. The minimum central anterior chamber depth must exceed 2.50 mm, the endothelial cell density threshold at 1600 cells/mm^2^, intraocular pressure below 22 mmHg, and bilateral corrected distance visual acuity result between 20/80 and 20/800 on the ETDRS chart. Patients should also be assessed for their cognitive capabilities and motivation to undergo a demanding visual rehabilitation program.

We herein present a case of a novel one-point scleral fixation of the IMT telescope.

## 2. Case Presentation

A 76-year-old female patient presented to Warsaw Eye Hospital with bilateral geographic atrophy and cataract. On examination, visual acuity measured with the ETDRS scale was 13 letters in the right eye and 15 letters in the left eye. Anterior segment examination revealed nuclear cataract in both eyes. Funduscopy showed geographic atrophy in both eyes. The patient was informed about the possibility of implanting the SIMG IMT telescope and agreed to the procedure. Anatomical parameters for IMT implantation were checked and met. Left eye endothelial cell density (ECD) count was 2759/mm^2^ centrally.

Uncomplicated phacoemulsification was performed using the EVA machine by DORC BV (Zuidland, the Netherlands). The 3 and 9 o’clock site ports and 2.2 mm main port were sealed after the lens removal, and an 8 mm scleral tunnel for SING IMT implantation was performed 1.5 mm away, parallel to the limbus. The device was implanted into the posterior capsule in the lower hemisphere, and two lower haptics were positioned intracapsularly at 5 and 7 o’clock. After successful placement of the lower haptics, the instability of the posterior bag was noted.

**Figure 1:**
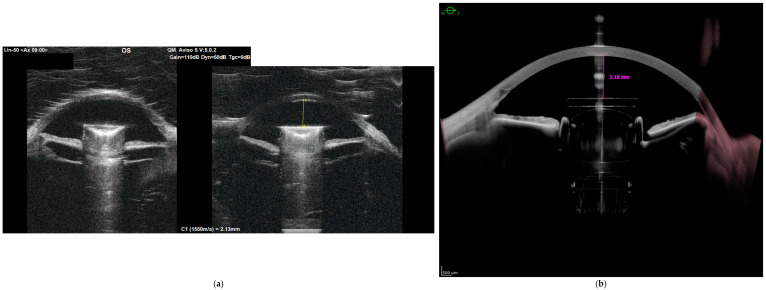
Ultrabiomicroscopy (**a**) and anterior segment optical coherence tomography (**b**) examinations showing proper positioning of the IMT telescope.

Upper haptic of IMT telescope situated at 12 o’clock was immediately positioned on the iris. At this point, removal of the IMT telescope from the eye or fixation to the sclera were the choices to follow. It was decided to suture the superior haptic of the IMT telescope on the 12 o’clock position and save the explanting possibility in case of scleral fixation failure. Due to the higher weight of the IMT telescope, it was decided to use 5-0, not 10-0, Prolene suture. The needle was passed through the middle section of the 12 o’clock haptic, and the suture was tightened to create the loop on the haptic part of the IMT. Consequently, the suture was placed through the sclera at a 12 o’clock position 1.5 mm away from the limbus. The length of the fixating suture was adjusted to allow IMT to be positioned in the center of the pupil. The suture was secured to the sclera by a knot which was partially buried in the scleral tunnel. At the end of the surgery, bulbar conjunctiva continuity was restored on the top of the scleral tunnel. The anterior chamber was checked for any vitreous presence. Partial vitrectomy was performed in the anterior chamber through the site port with a 23 Gauge vitrectomy probe. At the end of the surgery, the IMT telescope was placed properly in the eye with its optical part in the middle of the pupil. There was no vitreous present in the anterior chamber. On the first postoperative day, the eye looked quiet in the slit lamp, and IMT was positioned centrally. At 1 month post-operatively, stability was assessed through additional examinations (Figure 1a,b).

The patient was monitored for a duration of 18 months. Her visual parameters and positioning of the implant were monitored, and the results are described below. The postoperative medication regimen was typical for cataract surgery and included eye drops with tobramycin 4× daily for 7 days, bromfenac 2× daily for 6 weeks, and dexamethasone 4× daily for 2 weeks, the latter being reduced to 2× daily for the following 2 weeks. ECD measured in the center of the left cornea was 2541/mm^2^ at 1 month and 2446/mm^2^ at 18 months. After attending several visual rehabilitation sessions, the patient’s best corrected visual acuity in the operated eye reached 20 letters on ETDRS at 18 months.

## 3. Discussion

In 2023, the Food and Drug Administration (FDA) approved pegcetacoplan and avacincaptad pegol for the treatment of geographic atrophy [4]. They are inhibitors of the complement proteins C3 and C5, respectively, which are part of the immune system responsible for intensifying the inflammatory response. Complement activation results from aging of the retinal pigment epithelium (RPE), which, by reducing the retina’s protection from oxidative stress, causes chronic inflammation [5]. These drugs are intended to slow the gradual apoptosis of photoreceptors, especially before foveal involvement, but do not cause recovery of the damaged retina. Unfortunately, the results of the treatment so far have not been too promising [6]. As the disease progresses, conservative therapeutic options are limited to the use of magnifying glasses.

The new-generation implantable miniature telescope with a smaller incision (SING IMT™, Samsara Vision, Inc., New Jersey, USA) is intended for patients with bilateral advanced AMD. It is a seldom-used device due to its relatively high cost. Although, as in the case of refractive surgery in patients with healthy retinas, it was demonstrated that optical correction in the cornea or crystalline lens replacement provided better quality of vision than the use of eyeglasses, which do not cover the full field of vision and their position varies slightly on the nose [7].

In the initial stage, a phacoemulsification procedure is performed for the cataract with a 6 mm diameter capsulorhexis, according to the manufacturer’s recommendations. The preloaded implant is placed in the lens capsule through a scleral tunnel created by an 8 mm wide incision of the bulbar conjunctiva, made at the 12 o’clock position, slightly above the corneal limbus. After the implant haptics are properly positioned, the conjunctival wound is secured with sutures. The device is designed to magnify the image incident on the retina, enabling image perception with photoreceptors located perifoveally. Therefore, details from central vision reach the peripheral retina, effectively narrowing the field of view. This is the reason for implanting only one eye to preserve peripheral vision while maintaining binocular vision.

A study assessed best-corrected visual acuity for distance and near vision, loss of endothelial cell density, and the incidence of complications [8]. At three months postoperatively, the study eyes achieved an average improvement of +14.9 ± 7.1 letters, approximately 25% of the way to four or more rows on the ETDRS (Early Treatment Diabetic Retinopathy Study) charts, and in more than half of the patients by at least three rows. The beneficial effect of removing the cloudy lens should also be emphasized. The loss of endothelial cell density averaged 10.4 ±13.3% cells/mm^2^ after 3 months, which is similar to statistics reported after cataract surgery in patients from developed countries. The most frequently observed complication was corneal edema, but the effect of the method on postoperative astigmatism was not assessed.

The advantage of SING IMT™ over the Scharioth lens, which has been available on the market for over a decade, is its ability to magnify the image not only for near but also for distance, and also reduces the induction of optical aberrations that can distort the perceived image [9,10]. However, a significant disadvantage is the long neuroadaptation process, which can take up to 6–12 months [11,12]. To serve its purpose, the IMT telescope needs to be perfectly fixated in the eye within the posterior bag. Any instability will definitely influence its function, and severe instability could cause the device to luxate into the eye, which would probably be sight-threatening given the relatively large foreign body.

During the surgery described in this paper, it was decided to fixate only one upper haptic since the other two were placed within the capsular bag. This way, the number of additional steps that prolong intraocular surgery was minimized, decreasing the risk of postoperative complications. We did not use the scleral pocket to bury the suture since the location of the fixed haptic allowed the knot to be partially covered in the already existing scleral tunnel. Another case of scleral IMT fixation was described by Savastano et al. [13]. In their case, Gore-Tex suture was used for scleral fixation 90 days after the initial surgery of SING IMT implantation. In our case, however, the scleral fixation was performed during the initial procedure once the capsular instability was noted.

Scleral suturing of the SING IMT telescope was described for the first time by Eter and Behr in March 2024 [14]; however, the described technique was different. IMT telescope was sutured in two locations with 10-0 Prolene sutures. In our case, we decided to use thicker sutures since the increased weight of the IMT telescope would create an additional factor for suture breakage over the long follow-up period. It was reported that a 10-0 Prolene suture could dissolve after more than 10 years, adding to the instability of the fixed implant [15,16]. This was the case in several reports of children operated for congenital and early-onset cataract that had a subluxated implant years later in adulthood, with visual deterioration and the need for surgical repositioning. On the contrary, reports regarding the use of intraocular Prolene 5-0 affirm the reliable stability of IOLs [17,18]. Due to the very limited use of ophthalmic microtelescopes globally, let alone sutural fixation of IMT, the choice of suture was justified by the experienced surgeon’s intraoperative judgment of the tensile forces on the haptic and estimated load on the suture.

## 4. Conclusions

To the best of our knowledge, this is the first report of suturing the IMT Sing telescope to the sclera at one point using a 5-0 Prolene suture. Of course, further reports with longer follow-up are needed in order to determine the optimal technique in such challenging cases. However due to tilting often occurring in time after the implantation procedure, it would be relevant to standardize an approach to maintain the device stability in the long term, since proper positioning affects not only prevention of intraocular complications, but also speeds up neuroadaptation and impacts the quality of vision, which is particularly important in advanced AMD patients due to much lower visual potential of the perifoveal macula.

## Data Availability

The data presented in this study are available on reasonable request from the corresponding author.
